# Associations of burnout with job demands/resources during the pandemic in health workers from Southeast European countries

**DOI:** 10.3389/fpsyg.2023.1258226

**Published:** 2023-10-24

**Authors:** Dragan Mijakoski, Aneta Atanasovska, Dragana Bislimovska, Hana Brborović, Ognjen Brborović, Ljiljana Cvjeanov Kezunović, Milan Milošević, Jordan Minov, Buhara Önal, Nurka Pranjić, Liliana Rapas, Sasho Stoleski, Katya Vangelova, Roko Žaja, Petar Bulat, Aleksandar Milovanović, Jovanka Karadžinska-Bislimovska

**Affiliations:** ^1^Faculty of Medicine, Institute of Occupational Health of RN Macedonia, World Health Organization Collaborating Center (WHO CC), Global Allergy and Asthma Excellence Network Collaborating Center (GA2LEN CC), Ss. Cyril and Methodius University in Skopje, Skopje, North Macedonia; ^2^Department of Environmental and Occupational Health and Sports Medicine, School of Medicine, Andrija Štampar School of Public Health, University of Zagreb, Zagreb, Croatia; ^3^Department of Social Medicine and Organization of Health Care, School of Medicine, Andrija Štampar School of Public Health, University of Zagreb, Zagreb, Croatia; ^4^Department of Family Medicine, Medical Faculty Podgorica, University of Montenegro, Podgorica, Montenegro; ^5^International Commission on Occupational Health National Secretary (ICOH NS) for Turkey, Ankara, Türkiye; ^6^Department of Occupational Medicine, Medical Faculty, University of Tuzla, Tuzla, Bosnia and Herzegovina; ^7^Directorate of Public Health Bucharest, Ministry of Health, Bucharest, Romania; ^8^Department of Health at Work, National Center of Public Health and Analyses, Sofia, Bulgaria; ^9^Faculty of Medicine, Serbian Institute of Occupational Health, University of Belgrade, Belgrade, Serbia

**Keywords:** health workers, pandemic, occupational health, burnout, job demands

## Abstract

**Introduction:**

Despite several studies assessing job demands and burnout in countries from the Southeast European (SEE) region, there is still a lack of data about the psychological impact of the pandemic on health workers (HWs).

**Aims:**

The present study aimed to demonstrate and compare levels of burnout dimensions in HWs from SEE countries and to reveal the burnout–job demands/resources relationships in these workers during the pandemic.

**Materials and methods:**

During the autumn of 2020, this online multicentric cross-sectional survey studied a large group (*N* = 4.621) of HWs working in SEE countries. The Maslach Burnout Inventory was used for the measurement of burnout dimensions. We analyzed the job demands by using the Hospital Experience Scale. Remuneration and relationships with superiors were measured using the Questionnaire sur les Ressources et Contraintes Professionnelles (English version).

**Results:**

A series of ANOVA comparisons of means revealed the countries in which respondents showed higher mean values of emotional exhaustion (Bosnia and Herzegovina, Bulgaria, Croatia, Moldova, Montenegro, and North Macedonia) and the countries in which respondents showed lower mean values of this burnout dimension (Israel and Romania) (Welch *F* = 17.98, *p* < 0.001). We also found differences among HWs from different countries in job demands and job resources. The testing of hierarchical regression models, which have been controlled for certain confounding factors, clearly revealed that emotional exhaustion was predicted by job demands (*R*^2^ = 0.37) and job resources (*R*^2^ = 0.16).

**Conclusion:**

Preventive measures for the improvement of mental health in HWs during the pandemic and beyond have to take into account the differences between countries regarding the country context and current scientific knowledge. A modified stress test should be implemented in hospitals regarding future shocks that might include new pandemics, terrorism, catastrophes, or border conflicts.

## 1. Introduction

Health workers (HWs) are key stakeholders in delivering healthcare to patients that must be safe, timely, patient-centered, equitable, and effective. Long working hours, working under pressure with patients with coronavirus disease 2019 (COVID-19) infection, and having reduced rest periods during the COVID-19 pandemic put HWs at the front of the battle and thus at an increased risk of infection, resulting in psychological reactions such as chronic fatigue, anxiety, desolation, feelings of helplessness, and depression, among others (British Medical Association, 2020). Additionally, there has been significant disruption to usual practices, with many HWs being sent out of their usual workplaces and reassigned to work in more risky frontline positions.

Furthermore, the constant presence of fear of spreading the disease and the feeling of ‘no one is safe’, the concern over the availability of adequate personal protective equipment (PPE), discomfort caused by PPE usage, frequent changes of regulations, stigmatization in the community, and anger and aggression from patients and their families may result in chronic psychological distress, thus affecting the mental health of HWs. Therefore, the workplace environment that is rapidly changing during the ongoing emergency significantly increases the occupational risk to HWs (Cheng et al., 2020; De Kock et al., 2021; Sun et al., 2021).

Studies of the severe acute respiratory syndrome (SARS) and Middle East respiratory syndrome (MERS) outbreaks reported that HWs more frequently suffer from psychological distress (including anxiety, depression, and stigmatization) and also long-lasting mental health consequences that can develop even years after the beginning of an outbreak (Mousavi et al., 2021). Moreover, during these outbreaks, an estimated one-third of HWs were found to suffer from burnout syndrome (Magnavita et al., 2021).

Burnout relates to “exhaustion due to prolonged exposure to work-related problems” (Guseva Canu et al., 2021; Shoman et al., 2021). Maslach defines emotional exhaustion (an unbearable exhaustion of physical and emotional strengths and feelings of overload caused by workplace stressors or job demands), depersonalization (an indifferent and cynical behavior toward the job), and reduced personal accomplishment (when the worker feels incompetence and has low performance at the workplace) as three basic components of the syndrome (Maslach and Jackson, 1981; Maslach and Leiter, 1997; Maslach et al., 2001). In HWs, chronic emotional and interpersonal workplace stressors, together with exposure to additional workplace hazards, could result in the development of burnout symptoms.

Apart from the everyday workload and individual traits, such as perfectionism and difficulty coping with stress, the context of the pandemic, especially working with infected patients, and lack of resources, such as lack of training or good organizational rules, put additional strain on HWs (Preti et al., 2020; Meira-Silva et al., 2022). On the other hand, cooperative teamwork and good organizational support can increase job satisfaction and the delivery of safe and efficient patient care (Mijakoski et al., 2015a).

All these psychological changes and effects among HWs during the pandemic support the theoretical model that was proposed by Demerouti et al. (2001) (job demands–resources model), according to which burnout syndrome and its development in healthcare settings are caused by the presence of high job demands (all prolonged physical and/or psychological efforts of work), which cause overburdening and emotional exhaustion, and the lack of job resources, at both the organizational and interpersonal level, causing withdrawal behavior and disengagement (Mijakoski et al., 2015b).

Job satisfaction and working conditions directly influence the quality of care HWs provide to patients. Sufficient job resources are required, even in conditions in which job demands are high (such as during a pandemic); hence, the motivation and engagement for work among HWs could be sustained and burnout levels reduced (Gómez-Salgado et al., 2019; Thapa et al., 2022). Many studies have indicated that supervisor support and remuneration could influence career commitment in HWs, leading to improved work engagement and well-being, so they should not be neglected (Bertone and Witter, 2015; Hämmig, 2017; Xu et al., 2021; Heyns et al., 2022).

In the latest report from the World Innovation Summit for Health (WISH), supported by the WHO, around 40% of HWs were found to develop anxiety and depression, while the occurrence of burnout symptoms and their manifestations has outlined that younger HWs face higher mental health risks during times of pandemic. The psychological burden and rise of negative mental health impacts caused by this global health emergency have caused the so-called ‘pandemic within a pandemic’ (World Health Organization, 2022).

Previous studies that determine work demands and burnout dimensions in countries from the Southeast European (SEE) region exist. Differences in burnout between nurses and doctors working in hospital settings have been assessed (Mijakoski et al., 2015c), but there is a lack of data assessing HWs and the psychological impact that the pandemic had on them. Therefore, the present study aimed to demonstrate and compare levels of burnout dimensions among HWs from SEE countries and to reveal the burnout and job demands/resources relationships among these workers during the pandemic.

## 2. Materials and methods

### 2.1. Procedure

This study is a part of an online Survey titled “Job Stress in Health Workers during COVID-19 Pandemic,” conducted in SEE countries (Albania, Bosnia and Herzegovina, Bulgaria, Croatia, Israel, Moldova, Montenegro, North Macedonia, Romania, Serbia, and Turkey) during the autumn of 2020 by the SEE Network on Workers’ Health (SEENWH) with the SEE Health Network (Mijakoski et al., 2022). The coordinator of the activities was the Institute of Occupational Health of RN Macedonia, WHO CC in Skopje, North Macedonia. This multicentric cross-sectional survey studied a large group (*N* = 4.621) of HWs working in SEE countries. The majority of participants were female (78.4%). Their average age was 43.7 ± 10.7 years. The mean tenure was 18.8 ± 11.4 years. Participants were invited via e-mail, and links to the online questionnaires were available on the websites of the Medical and Nursing Chambers (for each of the participating countries) and through Microsoft Forms and LinkedIn. The participation was online, anonymous, and voluntary.

### 2.2. Survey instruments

The Maslach Burnout Inventory (MBI), as one of the most popular and validated burnout questionnaires, was applied for the measurement of burnout dimensions (Maslach et al., 2001). The MBI demonstrated high reliability for emotional exhaustion (*α* = 0.92) and depersonalization (*α* = 0.78). Nine items for emotional exhaustion and five items for depersonalization were scored using a 7-point Likert scale (0 = never to 6 = every day). Low emotional resources, reduced energy, feelings of unbearable exhaustion, and feelings of being used up were described as emotional exhaustion. Detached responses to other people, feelings of frustration, and cynicism defined the interpersonal dimension of burnout - depersonalization. The score for each dimension was calculated by adding responses, and each participant had separate scores for the two burnout components (Maslach et al., 2001).

The Hospital Experience Scale (HES) was applied for the evaluation of job demands. The HES was developed within the FP7 ORCAB Project by using qualitative thematic analysis (Montgomery et al., 2015; European Commission, 2022). Aimed at understanding workplace stressors in HWs, focus groups were organized with doctors, nurses, residents, and interns. The focus groups’ data were evaluated by thematic analysis, which revealed workplace stressors among HWs. Furthermore, these stressors were used for the development of HES items, which were additionally categorized into four subscales: physical (seven items, *α* = 0.73), organizational (six items, *α* = 0.79), emotional (six items, *α* = 0.75), and cognitive (five items, *α* = 0.69) job demands. More comprehensive data about the validation of the HES could be obtained upon request. HWs marked their level of agreement with the items using a 5-point Likert scale (1 = never to 5 = always). For different job demand subscales, a mean score was calculated (Montgomery et al., 2015).

Remuneration and Relationship with superior were analyzed using the Questionnaire sur les Ressources et Contraintes Professionnelles (QRCP) (English version). This instrument is developed based on the Questionnaire on the Experience and Assessment of Work (QEAW) (Lequeurre et al., 2013). Remuneration evaluates the approach workers reflect on their salary (Demerouti et al., 2001; Lequeurre et al., 2013). Relationship with superior demonstrates the relationships of workers with their superiors and the social support that employees could obtain from them (Bakker et al., 2008; Lequeurre et al., 2013). Remuneration consists of five items (*α* =0.82), and Relationship with superior (*α* =0.92) consists of 9 items. A 7-point Likert scale (from 1 = never to 7 = always) was used for the ratings on the Relationship with superior items, while Remuneration ranged from 1 = strongly disagree to 7 = strongly agree (Lequeurre et al., 2013). The MBI and HEs have translated and back-translated to every language of the SEE countries, validated for Croatian and Macedonian use. For the other languages, validation is ongoing.

### 2.3. Ethical permission

The SEENWH and Ethical Boards of the Institute of Occupational Health of RN Macedonia, the coordinator of the Project, approved the ethical issues of the study. Participants’ consent was requested and received.

### 2.4. Statistical analysis

The answers from all questionnaires were entered into an electronic database together with a check of the completeness of the data. Before other statistical analyzes, normality tests (reviewing histograms, skewness, kurtosis, box plot, and P–P Plot) were conducted. Where appropriate and necessary, a square root transformation of the data was applied. Taking into account the differences in sample sizes, Hochberg’s GT2 *post hoc* procedure was applied within ANOVA comparisons of means between several samples. We evaluated the correlation coefficients for each pair of predictor variables to check the possible multicollinearity between variables. We took into account that if the correlation coefficient was 0.8 or above, only one of the variables should have been used in the regression analysis. Multicollinearity was assessed using variance inflation factors (VIFs). We considered a VIF exceeding 5.0 as an indicator of multicollinearity. None of the VIF values for the predictor variables in this study were greater than 5, which showed that multicollinearity was not an issue in the regression models. Initially, bivariate analyzes were used to analyze the relationships of burnout dimensions with job demands, and job resources. Furthermore, the role of job demands and job resources for burnout dimensions was assessed by testing separate hierarchical multiple regression models for each burnout dimension. The models were controlled for sex, age, tenure, working hours per week, night-shift work, and contact with COVID-19 patients. In the first step, we entered age, tenure, working hours per week, night-shift work, and contact with COVID-19 patients, while in the second step, we entered job demands (or job resources).

## 3. Results

### 3.1. Participants

Completed surveys were returned by 4.621 HWs working in the countries of the SEE region (Albania 0.6%, Bosnia and Herzegovina 3.5%, Bulgaria 2.6%, Croatia 6.5%, Israel 0.5%, Moldova 5.2%, Montenegro 3.2%, North Macedonia 17.4%, Romania 55.2%, Serbia 1.6%, Turkey 3.4%, and other countries 0.4%). The majority of the participants were female (78.4%). This sex distribution was similar in the respective countries included in the survey. Their mean age was 43.7 ± 10.7 years, and they had worked for an average of 18.8 ± 11.4 years. Due to the small number of participants from Albania (*n* = 26), Israel (*n* = 24), and other countries (*n* = 20), the findings for these countries were analyzed with certain caution. The most frequent education level of the respondents was a university degree (4 years or more; *n* = 2.445, 52.9%), followed by a master’s/PhD (*n* = 1.076, 23.3%), bachelor’s (3 years; *n* = 786, 17%), and high school (*n* = 308, 6.7%) degree. The frequency distribution of the participants showed that they were specialist medical doctors (*n* = 1.779, 38.5%), nurses/technicians (*n* = 1.095, 23.7%), medical doctors (*n* = 904, 19.6%), nurses/technicians with bachelor’s degrees (*n* = 571, 12.4%), dentists (*n* = 111, 2.4%), and pharmacists (*n* = 32, 0.7%). They had worked in a public (*n* = 3.633, 78.6%) or private (*n* = 988, 21.4%) healthcare institution.

Of all the respondents, 2.184 (47.3%) reported night-shift work. Less than half (*n* = 2.009, 43.5%) of the participants answered that they had not had any occupational contact with self-isolated patients or patients who were positive for COVID-19, while 2.835 (61.4%) reported that HWs suffered a stigma as someone who could transmit the COVID-19 infection. The distribution of participants according to their overall satisfaction with the work in their institution was (ranging from 1 as the lowest level of job satisfaction to 5 as the highest level of job satisfaction) 1–191 (4.1%), 2–445 (9.6%), 3–1.467 (31.7%), 4–1.737 (37.6%), and 5–781 (16.9%).

### 3.2. Differences between see countries according to analyzed variables

A series of ANOVA comparisons of means revealed the countries in which respondents showed higher mean values of emotional exhaustion (Bosnia and Herzegovina, Bulgaria, Croatia, Moldova, Montenegro, and North Macedonia) and countries in which respondents showed lower mean values of this burnout dimension (Israel and Romania; Welch *F* = 17.98, *p* < 0.001). Analyzes also demonstrated the countries whose respondents showed higher mean values of depersonalization (Bosnia and Herzegovina, Moldova, North Macedonia, Croatia, and Turkey) and countries whose respondents showed lower mean values of this burnout dimension (Israel, Serbia, and Romania; Welch *F* = 16.54, *p* < 0.001; see Table 1).

**Table 1:** Descriptive statistics of burnout dimensions and differences between SEE countries.

	Country	Mean	SD	SE	Welch *F* (*p*)
Burnout–Emotional Exhaustion	Albania	21.7	14.8	2.9	17.98 (<0.001)
	Bosnia and Herzegovina	21.9	12.4	0.9	
	Bulgaria	23.8	13.5	1.2	
	Croatia	23.7	12.2	0.7	
	Israel	13	10.9	2.2	
	Moldova	22.9	13.2	0.9	
	Montenegro	22.4	13.3	1.1	
	North Macedonia	24.1	12.8	0.5	
	Romania	18.2	12.6	0.2	
	Serbia	20.1	13.9	1.6	
	Turkey	20.4	15.02	1.2	
	Other countries	15.8	11.9	2.7	
Burnout–Depersonalization	Albania	4.3	6.2	1.2	16.54 (<0.001)
	Bosnia and Herzegovina	5.9	5.6	0.4	
	Bulgaria	5.5	5.7	0.5	
	Croatia	6.8	6	0.3	
	Israel	2.7	2.4	0.5	
	Moldova	6.6	6.9	0.4	
	Montenegro	5.7	6.8	0.6	
	North Macedonia	6.2	5.9	0.2	
	Romania	4.1	4.9	0.1	
	Serbia	3.6	5.8	0.7	
	Turkey	6.8	7.4	0.6	
	Other countries	5	6	1.3	

Since there are no standards or criteria for the categorization of samples into high-, medium-, or low-demand groups, for this study we have used the findings of ANOVA comparisons between groups for categorizing countries into different levels of analyzed variables. Three groups of countries were detected according to the mean values of physical job demands: countries in which respondents showed higher mean values of physical job demands (Bosnia and Herzegovina and Croatia), countries with medium mean values of physical job demands (Albania, Bulgaria, Moldova, Montenegro, North Macedonia, Romania, and Serbia), and countries with lower mean values of physical job demands (Israel and Turkey; Welch *F* = 30.6, *p* < 0.001). Three groups of countries were also detected according to the mean values of organizational job demands: countries with higher mean values (Bosnia and Herzegovina and Croatia), countries with medium mean values (Albania, Bulgaria, Israel, Moldova, Montenegro, North Macedonia, Romania, Serbia, and Turkey), and countries with lower mean values of organizational job demands (other countries; Welch *F* = 12.09, *p* < 0.001). According to the mean values of emotional job demands, these three groups of countries were detected: countries with higher mean values (Bosnia and Herzegovina and Croatia), countries with medium mean values (Albania, Bulgaria, Moldova, Montenegro, North Macedonia, Romania, Serbia, and Turkey), and countries with lower mean values of emotional job demands (Israel and other countries; Welch *F* = 12.5, *p* < 0.001). According to the mean values of cognitive job demands, three groups of countries were detected: countries with higher mean values (Albania, Bosnia and Herzegovina, and Croatia), countries with medium mean values (Montenegro, North Macedonia, and Serbia), and countries with lower mean values of cognitive job demands (Bulgaria, Israel, Moldova, Romania, Turkey, and other countries; Welch *F* = 21.65, *p* < 0.001; see Table 2).

**Table 2:** Descriptive statistics of job demands and differences between SEE countries.

	Country	Mean	SD	SE	Welch *F* (*p*)
Physical Job Demands	Albania	3.3	0.9	0.2	30.6 (<0.001)
	Bosnia and Herzegovina	3.8	0.7	0.1	
	Bulgaria	3.6	0.6	0.1	
	Croatia	3.9	0.6	0.04	
	Israel	2.7	0.9	0.2	
	Moldova	3.6	0.7	0.04	
	Montenegro	3.6	0.7	0.1	
	North Macedonia	3.5	0.7	0.03	
	Romania	3.4	0.7	0.01	
	Serbia	3.4	0.8	0.1	
	Turkey	2.7	0.8	0.1	
	Other countries	3.1	0.7	0.2	
Organizational Job Demands	Albania	2.6	1.2	0.2	12.09 (<0.001)
	Bosnia and Herzegovina	3.2	0.8	0.1	
	Bulgaria	2.7	0.8	0.1	
	Croatia	3.2	0.8	0.05	
	Israel	2.6	0.9	0.2	
	Moldova	2.7	0.8	0.1	
	Montenegro	2.9	0.8	0.1	
	North Macedonia	2.8	0.9	0.03	
	Romania	2.9	0.8	0.02	
	Serbia	2.7	0.8	0.1	
	Turkey	2.6	1.1	0.1	
	Other countries	2.3	0.8	0.2	
Emotional Job Demands	Albania	2.4	0.9	0.2	12.5 (<0.001)
	Bosnia and Herzegovina	2.7	0.7	0.1	
	Bulgaria	2.4	0.7	0.1	
	Croatia	2.7	0.7	0.04	
	Israel	2.1	0.6	0.1	
	Moldova	2.6	0.7	0.05	
	Montenegro	2.5	0.7	0.1	
	North Macedonia	2.6	0.7	0.02	
	Romania	2.4	0.7	0.01	
	Serbia	2.4	0.8	0.1	
	Turkey	2.5	0.9	0.1	
	Other countries	2.3	0.7	0.2	
Cognitive Job Demands	Albania	2.9	1.1	0.2	21.65 (<0.001)
	Bosnia and Herzegovina	2.9	0.8	0.1	
	Bulgaria	2.6	0.8	0.1	
	Croatia	3.1	0.7	0.04	
	Israel	2.5	0.7	0.1	
	Moldova	2.6	0.8	0.05	
	Montenegro	2.7	0.8	0.1	
	North Macedonia	2.8	0.8	0.03	
	Romania	2.5	0.8	0.02	
	Serbia	2.7	0.9	0.1	
	Turkey	2.5	0.9	0.1	
	Other countries	2.5	0.6	0.1	

Three groups of countries were detected according to the mean values of the job resource Remuneration: countries in which respondents showed higher mean values of Remuneration (Romania and other countries), countries with medium mean values of Remuneration (Albania, Bosnia and Herzegovina, Bulgaria, Croatia, N Macedonia, Serbia, and Turkey), and countries with lower mean values of Remuneration (Israel, Moldova, and Montenegro; Welch *F* = 49.23, *p* < 0.001). Three groups of countries were also detected according to the mean values of the job resource Supervisor support: countries with higher mean values (Albania, Bulgaria, Israel, Moldova, and other countries), countries with medium mean values (Bosnia and Herzegovina, Montenegro, North Macedonia, and Romania), and countries with lower mean values (Croatia, Serbia, and Turkey; Welch *F* = 10.15, *p* < 0.001; see Table 3).

**Table 3:** Descriptive statistics of job resources and differences between SEE countries.

	Country	Mean	SD	SE	Welch *F* (*p*)
Remuneration	Albania	2.7	1.1	0.2	49.23 (<0.001)
	Bosnia and Herzegovina	2.8	1	0.1	
	Bulgaria	2.6	1.1	0.1	
	Croatia	2.6	1	0.1	
	Israel	2.4	1.1	0.2	
	Moldova	2.4	0.9	0.1	
	Montenegro	2.2	1	0.1	
	North Macedonia	2.6	1.1	0.04	
	Romania	3.2	1	0.02	
	Serbia	2.5	0.9	0.1	
	Turkey	2.7	1.2	0.1	
	Other countries	3.8	0.8	0.2	
Supervisor support	Albania	3.9	0.9	0.2	10.15 (<0.001)
	Bosnia and Herzegovina	3.5	0.9	0.1	
	Bulgaria	4	1	0.1	
	Croatia	3.4	1	0.1	
	Israel	4	0.8	0.2	
	Moldova	4	0.7	0.04	
	Montenegro	3.6	0.9	0.1	
	North Macedonia	3.7	1	0.04	
	Romania	3.7	0.9	0.02	
	Serbia	3.4	1	0.1	
	Turkey	3.4	1.1	0.1	
	Other countries	4.1	0.8	0.2	

### 3.3. Independent predictors of burnout dimensions in the study sample

The bivariate analysis has shown a significant positive correlation between emotional exhaustion and depersonalization with physical, organizational, emotional, and cognitive job demands in study participants. We found that both burnout dimensions were negatively correlated with Remuneration and Supervisor support. Job resources were also negatively correlated with physical, organizational, emotional, and cognitive job demands (see Table 4).

**Table 4:** Correlations of analyzed variables.

	1	2	3	4	5	6	7	8
1. Emotional Exhaustion	0.92							
2. Depersonalization	0.636*	0.779						
3. Physical job demands	0.446*	0.298*	0.739					
4. Organizational job demands	0.444*	0.357*	0.547*	0.762				
5. Emotional job demands	0.541*	0.465*	0.448*	0.555*	0.678			
6. Cognitive job demands	0.478*	0.393*	0.507*	0.539*	0.583*	0.69		
7. Remuneration	−0.283*	−0.175*	−0.281*	−0.251*	−0.211*	−0.214*	0.905	
8. Supervisor support	−0.307*	−0.23*	−0.257*	−0.459*	−0.331*	−0.313*	0.299*	0.948

*Correlation is significant at the < 0.01 level (two-tailed). Cronbach’s alpha on the diagonal.

The standardized beta coefficients for the independent predictors (including job demands) of emotional exhaustion are presented in Table 5. We found that physical (*β* = 0.18, *p* < 0.01), organizational (*β* = 0.09, *p* < 0.01), emotional (*β* = 0.31, *p* < 0.01), and cognitive (*β* = 0.15, *p* < 0.01) job demands, working hours per week (*β* = 0.06, *p* < 0.05), and contact with patients with COVID-19 (*β* = 0.03, *p* < 0.05) were significant positive predictors of emotional exhaustion, whereas male sex (*β* = −0.03, *p* < 0.05) and working night shifts (*β* = −0.1, *p* < 0.01) negatively predicted emotional exhaustion (*R*^2^ for the model = 0.371).

**Table 5:** Hierarchical multiple regression model for emotional exhaustion including job demands.

Emotional Exhaustion		B	SE	95% CI for B		Beta	*R* ^2^
				Lower	Upper		
Step 1	Sex	−2.27	0.47	−3.19	−1.36	−0.07**	0.036
	Age	−0.03	0.05	−0.12	0.07	−0.02	
	Tenure	−0.04	0.04	−0.13	0.05	−0.04	
	Working hours per week	0.15	0.02	0.11	0.19	0.12**	
	Night-shift work	−1.93	0.42	−2.74	−1.11	−0.07**	
	Contact with COVID-19 patients	3.5	0.4	2.73	4.28	0.13**	
	Constant	14.94	1.6	11.8	18.09		
Step 2	Sex	−0.91	0.38	−1.65	−1.17	−0.03*	0.371
	Age	0.03	0.04	−0.05	0.1	0.02	
	Tenure	−0.05	0.04	−0.11	0.02	−0.04	
	Working hours per week	0.07	0.02	0.04	0.11	0.06**	
	Night-shift work	−2.66	0.34	−3.33	−2.01	−0.1**	
	Contact with COVID-19 patients	0.86	0.33	0.22	1.49	0.03**	
	Physical Job Demands	3.05	0.26	2.54	3.57	0.18**	
	Organizational Job Demands	1.46	0.25	0.97	1.94	0.09**	
	Emotional Job Demands	5.61	0.28	5.06	6.17	0.31**	
	Cognitive Job Demands	2.51	0.26	2.01	3.01	0.15**	
	Constant	−17.56	1.49	−20.48	−14.65		

*R*^2^ = 0.036 for Step 1; Δ*R*^2^ = 0.335 for Step 2 (*p* < 0.001). *p < 0.05, **p < 0.01.

The standardized beta coefficients for the independent predictors (including job resources) of emotional exhaustion are shown in Table 6. We demonstrated that remuneration (*β* = −0.2, *p* < 0.01), supervisor support (*β* = −0.24, *p* < 0.01), male sex (*β* = −0.07, *p* < 0.01), and working night shifts (*β* = −0.06, *p* < 0.01) negatively predicted emotional exhaustion, while the number of working hours per week (*β* = 0.1, *p* < 0.01) and contact with patients with COVID-19 (*β* = 0.11, *p* < 0.01) were significant positive predictors of emotional exhaustion (*R*^2^ for the model = 0.161).

**Table 6:** Hierarchical multiple regression model for emotional exhaustion including job resources.

Emotional Exhaustion		B	SE	95% CI for B		Beta	*R* ^2^
				Lower	Upper		
Step 1	Sex	−2.27	0.47	−3.19	−1.36	−0.07*	0.036
	Age	−0.03	0.05	−0.12	0.07	−0.02	
	Tenure	−0.04	0.04	−0.13	0.05	−0.04	
	Working hours per week	0.15	0.02	0.11	0.19	0.12*	
	Night-shift work	−1.93	0.42	−2.74	−1.11	−0.07*	
	Contact with COVID-19 patients	3.5	0.4	2.73	4.28	0.13*	
	Constant	14.94	1.6	11.8	18.09		
Step 2	Sex	−2.11	0.43	−2.96	−1.26	−0.07*	0.161
	Age	0.001	0.04	−0.08	0.09	0.001	
	Tenure	−0.07	0.04	−0.15	0.01	−0.06	
	Working hours per week	0.13	0.02	0.09	0.16	0.1*	
	Night-shift work	−1.62	0.39	−2.38	−0.86	−0.06*	
	Contact with COVID-19 patients	2.77	0.37	2.04	3.5	0.11*	
	Remuneration	−2.52	0.18	−2.86	−2.17	−0.2*	
	Supervisor support	−3.24	0.2	−3.62	−2.85	−0.24*	
	Constant	34.94	1.7	31.6	38.27		

*R*^2^ = 0.036 for Step 1; Δ*R*^2^ = 0.125 for Step 2 (*p* < 0.001). *p < 0.01.

Within Table 7 we have presented the standardized beta coefficients for the independent predictors (including job demands) of depersonalization. The obtained data demonstrated that physical (*β* = 0.05, *p* < 0.01), organizational (*β* = 0.07, *p* < 0.01), emotional (*β* = 0.31, *p* < 0.01), and cognitive (*β* = 0.14, *p* < 0.01) job demands, male sex (*β* = 0.09, *p* < 0.01), and contact with patients with COVID-19 (*β* = 0.08, *p* < 0.01) were significant positive predictors of depersonalization, whereas working night shifts (*β* = −0.05, *p* < 0.01) was detected as a significant negative predictor of depersonalization (*R*^2^ for the model = 0.263).

**Table 7:** Hierarchical multiple regression model for depersonalization including job demands.

Depersonalization		B	SE	95% CI for B		Beta	*R* ^2^
				Lower	Upper		
Step 1	Sex	0.84	0.2	0.45	1.23	0.06**	0.046
	Age	−0.04	0.02	−0.08	0	−0.08*	
	Tenure	−0.02	0.02	−0.06	0.01	−0.05	
	Working hours per week	0.04	0.01	0.02	0.05	0.06**	
	Night-shift work	−0.41	0.18	−0.76	−0.06	−0.04*	
	Contact with COVID-19 patients	1.74	0.17	1.40	2.07	0.15**	
	Constant	4.58	0.69	3.22	5.93		
Step 2	Sex	1.22	0.18	0.87	1.56	0.09**	0.263
	Age	−0.03	0.02	−0.06	0.01	−0.05	
	Tenure	−0.02	0.02	−0.05	0.01	−0.04	
	Working hours per week	0.01	0.01	−0.01	0.03	0.02	
	Night-shift work	−0.58	0.16	−0.89	−0.27	−0.05**	
	Contact with COVID-19 patients	0.91	0.15	0.61	1.21	0.08**	
	Physical Job Demands	0.34	0.12	0.1	0.58	0.05**	
	Organizational Job Demands	0.5	0.12	0.27	0.73	0.07**	
	Emotional Job Demands	2.4	0.13	2.14	2.66	0.31**	
	Cognitive Job Demands	0.98	0.12	0.74	1.21	0.14**	
	Constant	−5.63	0.7	−7	−4.27		

*R*^2^ = 0.046 for Step 1; Δ*R*^2^ = 0.216 for Step 2 (*p* < 0.001). *p < 0.05, **p < 0.01.

The standardized beta coefficients for the independent predictors (including job resources) of depersonalization are presented in Table 8. We found that remuneration (*β* = −0.11, *p* < 0.01), supervisor support (*β* = −0.19, *p* < 0.01), and working night shifts (*β* = −0.03, *p* < 0.05) negatively predicted depersonalization, while male sex (*β* = 0.06, *p* < 0.01), number of working hours per week (*β* = 0.05, *p* < 0.01), and contact with patients with COVID-19 (*β* = 0.13, *p* < 0.01) were significant positive predictors of depersonalization (*R*^2^ for the model = 0.161).

**Table 8:** Hierarchical multiple regression model for depersonalization including job resources.

Depersonalization		B	SE	95% CI for B		Beta	*R* ^2^
				Lower	Upper		
Step 1	Sex	0.84	0.2	0.45	1.23	0.06**	0.046
	Age	−0.04	0.02	−0.08	0	−0.08*	
	Tenure	−0.02	0.2	−0.06	0.01	−0.05	
	Working hours per week	0.04	0.01	0.02	0.05	0.06**	
	Night-shift work	−0.41	0.18	−0.76	−0.06	−0.04*	
	Contact with COVID-19 patients	1.74	0.17	1.4	2.07	0.15**	
	Constant	4.58	0.69	3.22	5.93		
Step 2	Sex	0.88	0.19	0.5	1.26	0.06**	0.106
	Age	−0.04	0.02	−0.07	0.003	−0.07	
	Tenure	−0.03	0.02	−0.07	0.01	−0.06	
	Working hours per week	0.03	0.01	0.01	0.05	0.05**	
	Night-shift work	−0.35	0.17	−0.69	−0.01	−0.03*	
	Contact with COVID-19 patients	1.52	0.17	1.2	1.85	0.13**	
	Remuneration	−0.6	0.08	−0.75	−0.44	−0.11**	
	Supervisor support	−1.12	0.09	−1.29	−0.94	−0.19**	
	Constant	10.77	0.76	9.28	12.26		

*R*^2^ = 0.046 for Step 1; Δ*R*^2^ = 0.06 for Step 2 (*p* < 0.001). *p < 0.05, **p < 0.01.

## 4. Discussion

### 4.1. Burnout

Throughout the course of the COVID-19 pandemic, a significant number of healthcare workers (HWs) have experienced burnout, prompting numerous studies to investigate this phenomenon. According to the World Health Organization (WHO), there has been an estimated range of 80,000 to 180,000 HWs who have lost their lives globally as a result of the COVID-19 pandemic (Arbar, 2021). The majority of individuals in question were medical professionals, specifically doctors and nurses. Based on the findings of Orrù et al. (2021), it has been observed that a significant number of HWs lost their lives due to various factors related to psychological stress. These factors include but are not limited to uncertainties surrounding the progression of the disease, both in terms of short-term and long-term effects, and concerns about the efficacy of available treatments. Additionally, the lack of personal protective equipment (PPE) poses a significant challenge, further exacerbating the psychological burden on HWs. Physical exhaustion resulting from excessive workloads also contributes to the overall stress levels experienced by these individuals. Moreover, the fear of direct exposure to COVID-19 in the workplace adds to the psychological strain faced by HWs (Britt et al., 2021; Ferry et al., 2021).

In a study conducted by Ulfa et al. (2022), it was found that in a total of 48 countries, numerous publications have made significant contributions to the advancement of research on the burnout status of HWs. The geographical distribution of these countries encompasses the United States, Spain, China, Italy, Taiwan, France, Canada, Malaysia, South Korea, and the United Kingdom. This distribution sheds light on the scientific production exhibited by each respective country. According to the data collected, it has been observed that the United States (US) has emerged as the most productive country, with a total count of 26. Following closely behind is Spain, with a count of 20, and China, with a count of 17. These findings suggest that these three countries have demonstrated a significant level of productivity in the given context.

### 4.2. Sociodemographic data

In the current study, we obtained results from SEE countries (Albania 0.6%, Bosnia and Herzegovina 3.5%, Bulgaria 2.6%, Croatia 6.5%, Israel 0.5%, Moldova 5.2%, Montenegro 3.2%, North Macedonia 17.4%, Romania 55.2%, Serbia 1.6%, Turkey 3.4%, and other countries 0.4%). Due to the small number of participants from Albania (*n* = 26), Israel (*n* = 24), and other countries (*n* = 20), the findings for these countries were analyzed with certain caution.

The present study consisted of a predominantly female sample, accounting for over half of the participants (78.4%). The average age of the participants was 43.7 years, with a standard deviation of 10.7 years. Additionally, the average tenure of the participants was 18.8 years, with a standard deviation of 11.4 years. The findings of this study align with previous research, which has consistently shown that female workers make up the predominant proportion of the health workforce (Asamani et al., 2019; Dubale et al., 2019; Suleiman et al., 2020; Afulani et al., 2021).

### 4.3. Job demands

HWs who are actively engaged in the frontline management of patients diagnosed with COVID-19 face the potential risk of experiencing stigmatization. Among the participants in our study (*n* = 2,009), a minority of individuals (43.5%) indicated that they had not engaged in any occupational interactions with patients who were either self-isolated or tested positive for COVID-19. Conversely, a majority of respondents (61.4%) reported that healthcare professionals experienced stigmatization as potential transmitters of infection during the COVID-19 pandemic. There is an increasing body of evidence indicating that the stigma surrounding COVID-19 has emerged as a significant contributor to the mental distress experienced by frontline HWs and affected individuals. This distress manifests in various forms, including stress, anxiety, and depression, and has profound implications for their overall well-being (Bao et al., 2020; Gunnell et al., 2020; Peprah, 2020). The phenomenon of COVID-19-induced stigma has been observed to have a significant impact on individuals, particularly HWs, leading to feelings of isolation and diminished self-worth. This is primarily attributed to their perceived inability to make meaningful contributions to the ongoing battle against the pandemic (Bao et al., 2020; Holmes et al., 2020).

Among the entirety of the participants, it was observed that a total of 2.184 individuals, accounting for approximately 47.3% of the sample, indicated their engagement in night-shift employment. The night-shift workers in question are compelled to engage in work and rest patterns that are incongruous with their natural circadian rhythm. Accordingly, it has been postulated that disruptions to the circadian rhythm and sleep patterns may serve as plausible catalysts for the adverse consequences associated with night-shift employment, including the development of cardiovascular ailments and type 2 diabetes mellitus (Knutsson, 2003; Puttonen et al., 2010; Moreno et al., 2019). The potential relationship between sleep disrupted by circadian rhythm disturbances and the impact on the immune system has garnered significant attention among researchers. In light of this, there has been a growing interest in investigating the potential link between night-shift work and an increased vulnerability to infections (Almeida and Malheiro, 2016). Previous research has indicated a positive correlation between night-shift work and heightened incidences of common infections (Mohren et al., 2002; Prather and Carroll, 2021). Moreover, our research revealed that in a meticulously designed study, there was a notable disparity in the incidence of respiratory infections among healthcare workers engaged in night-shift work compared to their counterparts involved in day-shift work. Specifically, the former group exhibited a 20% higher incidence of respiratory infections (Loef et al., 2019). Nevertheless, the extent to which comparable outcomes can be anticipated beyond the realm of healthcare and concerning particular infection categories remains uncertain.

### 4.4. Job satisfaction

The present research has revealed a notable decline in the level of satisfaction experienced by healthcare workers (HWs) concerning their professional work. This decline can be attributed to the challenges and demands imposed on them as a result of their work during the COVID-19 pandemic. The data reveal the distribution of participants based on their overall satisfaction with their institution’s work. The satisfaction levels were measured on a scale of 1 to 5, with 1 representing the lowest level of satisfaction and 5 representing the highest level of satisfaction. The breakdown of participants across these satisfaction levels was as follows: 191 participants (4.1%) reported a satisfaction level of 1, 445 participants (9.6%) reported a satisfaction level of 2, 1,467 participants (31.7%) reported a satisfaction level of 3, 1,737 participants (37.6%) reported a satisfaction level of 4, and 781 participants (16.9%) reported a satisfaction level of 5. The research conducted by Abd-Ellatif et al. (2021) revealed that a significant proportion of participants, specifically 41.2%, reported experiencing a low level of job satisfaction. This decline in job satisfaction was primarily attributed to the fear of contracting infections amidst the ongoing pandemic (Abd-Ellatif et al., 2021). Based on the findings of another study, it was observed that among a group of nurses working on wards where care for individuals afflicted with COVID-19 is not provided, approximately 10% of respondents expressed a serious contemplation of transitioning to a different profession. Conversely, in wards where patients suffering from COVID-19 were being treated, a significantly higher proportion of nurses, specifically 24.8%, indicated their inclination toward changing their current occupation (Said and El-Shafei, 2021). Labrague and De Los Santos (2020) have highlighted the significance of the circumstances surrounding the necessity to operate under increasingly challenging conditions, which has resulted in a notable decline in job satisfaction among nursing personnel. Consequently, this has effectively influenced their inclination to pursue alternative professional paths (Labrague and De Los Santos, 2020; De Los Santos and Labrague, 2021). In order to determine the primary factor contributing to the decrease in job satisfaction among nursing personnel, Soto-Rubio et al. (2020) conducted a study examining the relationship between a pandemic and the prevalence of psychosocial risks. Their findings indicate a positive correlation between the two, specifically in terms of increased risk of accidents at work, low work commitment, and mental illness (Soto-Rubio et al., 2020). The adverse effects of diminished job satisfaction on the organizational commitment of healthcare workers (HWs) have been well documented. This phenomenon has been observed to potentially exacerbate staff shortages within healthcare organizations, and it is widely recognized as a primary driver behind the high turnover rates among medical professionals. Research has shown that employees who experience a high level of job satisfaction tend to exhibit greater levels of creativity, dedication, and engagement in their work. This positive relationship between employee satisfaction and these desirable work outcomes has been observed in various organizational contexts. Furthermore, the research that was conducted has revealed a clear and direct correlation between the level of satisfaction experienced by HWs and the level of satisfaction reported by patients concerning the care they received during their hospital stay (Akinwale and George, 2020). Research has shown that there is a positive correlation between employee satisfaction and workplace performance. Specifically, individuals who report higher levels of job satisfaction tend to exhibit higher levels of productivity in their respective work environments. This suggests that employee satisfaction plays a crucial role in fostering a more productive workforce. Hospital management must prioritize the cultivation of a high level of job satisfaction among their employees. By doing so, they can enhance work efficiency, ultimately leading to improved patient care (Karem et al., 2019).

### 4.5. Country-specific results, challenges during the pandemic, and remuneration

The majority of the participating SEE countries followed the WHO recommendations at the time this research was conducted, and similar events took place: (1) In the healthcare sector, triage stations at healthcare facility entrances and COVID departments were opened, protective infection measures were undertaken, and information on treatment and prevention was provided for HWs, following the recommendations of the WHO. In the autumn of 2020, HWs were provided with adequate high-quality personal protective equipment. Additionally, the health sector was temporary restructured by establishing COVID hospitals and by reorganizing the provision of medical services to the chronically ill; (2) along with the increase of patients, in 2020, many HWs were infected, which led to a further decrease of the staff and consequently an increase of the workload and working hours; (3) additional stressors during the pandemic were the high risk of being infected and/or transmitting the virus, fear of exposing family members, losing patients, emergency patients, high number of patients, high workload, time pressure, long working hours, need to practice outside of the area of expertise, treating co-workers, or personal and lifestyle stressors; (4) a period of negotiations and correspondence using digitized “socio-professional vehicles” (such as e-mails, media messages, and social networks) was established; (5) the disjunction between the “pandemic” and “working conditions” was manifested both in economic terms and legislatively until the harmonization of European laws (with SARS-COV-2 being included in the risk group 3 and COVID-19 included in the List of occupational diseases for HWs), the provision of appropriate protective equipment for interventions in outbreaks, and the approval of the vaccine; (6) the HWs were reimbursed for their work during the pandemic, receiving additional payment, but the reimbursement started later, after the survey was carried out; (7) the visibility of HWs faced with the uncertainty of the pandemic phenomenon made them either the target of admiration “heroes” or the target of stigma-related violence, harassment, and aggression generated by the frustration resulting from the isolation and quarantine measures and the limitations to mobility or work; and (8) working hours during the pandemic changed significantly from working 2 weeks (and resting the next 2 – the possible incubation period) to working 12-, 24-, or 48- h shifts.

The findings of a prior study conducted in Bosnia and Herzegovina revealed that a significant proportion, specifically 77%, of HWs in the country reported experiencing various manifestations of burnout amidst the ongoing pandemic. The findings of this study indicate that a significant proportion, specifically 32%, of the participants have experienced all three manifestations of burnout (Mijić Marić et al., 2022). The data collected from the respondents indicated a notable disparity between the levels of personal and work-related burnout when compared to the level of burnout specifically related to patient care. Healthcare workers have been subjected to stigmatization amidst the ongoing COVID-19 pandemic. The social stigma surrounding these individuals, characterized by fear and avoidance, has emerged as a prevalent and acknowledged issue across countries in Southeastern Europe (SEE) during this public health crisis. Bosnia and Herzegovina has demonstrated the highest degree of stigmatization, reaching a notable level of 82%. The study conducted in Bosnia and Herzegovina found a significant association between the perception of stigma among HWs and the perception of depersonalization (*p* = 0.002; Pranjic, 2021).

There are data on the widespread job stress and burnout among HWs before the pandemic in Bulgaria (Peev, 2017; Vangelova et al., 2019, 2021; Asenova et al., 2021). The study findings of higher mean values of emotional exhaustion within the sample of HWs from Bulgaria are consistent with our previous findings in a survey from 2018 showing high emotional exhaustion of hospital HWs, determined by time pressure, uncertainty, high strain, frustration, and lack of autonomy (Vangelova et al., 2019, 2021). Moreover, the long working hours were not a significant predictor of emotional exhaustion in our previous study; emotional exhaustion increased with the increase of night work and long working hours every week. Overtime and multiple workplaces are common in Bulgaria both for physicians and nurses, contributing to long weekly working hours (>41 h weekly for 80% of the studied physicians and 65.4% of the nurses, including >61 h weekly for 13.7% of the physicians and 13.4% nurses) (Vangelova et al., 2019, 2021). The medium mean values of physical job demands, organizational job demands, and emotional job demands are most probably due to the time the survey was conducted, in the autumn of 2020, which was a period with a comparatively low number of COVID cases. The medium mean values of remuneration with Bulgarian HCWs are well justified, taking into account the results of the previous study showing that a great deal of the studied physicians and nurses considered their payment unsatisfactory (Vangelova et al., 2019, 2021). In our previous studies (Vangelova et al., 2019, 2021), more than 70% of the physicians and 40% of the nurses had autonomy in their work, about 70% of both groups rated good opportunities for professional development, and about 40% considered there was justice in the distribution of work between the staff. The higher mean values for supervisor support are consistent with previous data from a study from 2018 conducted among hospital HWs (Vangelova et al., 2019, 2021).

From the onset of the pandemic, the Croatian Ministry of Health decided that all HWs were entitled to financial compensation while they were in isolation or at home (some HWs worked 2 weeks and had 2 weeks off afterward) and to compensation or days off work for working overtime (Ministry of Health, 2020). Shortly after this research took place, the government decided to increase the wages of HWs by 10% (Government of Republic of Croatia, 2020; Ministry of Health, 2020). High job demands were brought about by different changes in work organizations. The changes were frequent, following every new guideline that was brought by the government and the Croatian Institute of Public Health on a monthly or even weekly basis (Tokić et al., 2021). Emotional demands and burnout were associated with high job demands in our research and other Croatian research (Tokić et al., 2021). Some authors have reported changes in interpersonal relationships due to the pandemic and pandemic measures, which might contribute to emotional demands and burnout or working as a HW during the pandemic in general (Tokić et al., 2021; Šego, 2022).

In February 2020, North Macedonia officially reported its first-ever case of SARS-CoV-2, the causative agent of the ongoing coronavirus disease (COVID-19) pandemic. In late January, a series of initial national measures were implemented in response to the potential outbreak and treatment of a particular infectious disease. These measures included the installation of thermal cameras at the national airport and the provision of personal protective equipment and reagents for the detection of the virus. A sequence of public health measures and recommendations was adhered to. The escalating incidence of novel cases necessitated the implementation of enhanced and robust measures to effectively curb the propagation of the virus. The implementation of a comprehensive closure of educational institutions, ranging from kindergartens to universities, was initiated on March 10. On March 18, an official declaration of a state of emergency was made, signifying a critical situation requiring immediate action. Subsequently, on March 23, the first set of movement restrictions was implemented at the national level, aiming to regulate and limit the mobility of individuals within the affected area. The implementation of a curfew between the hours of 9 pm and 5 am on weekdays, along with specific measures targeting the elderly and individuals under the age of 18, was proven inadequate in effectively mitigating the transmission of the virus. Consequently, more stringent measures pertaining to the limitation of movement were implemented on April 8. These measures included a prohibition on movement between the hours of 4 pm and 5 am the following day and a complete ban on movement during weekends, commencing at 4 pm on Friday and concluding at 5 am on Monday. During the religious holidays in the country, namely, Orthodox Easter (17–21 April 2020) and Eid al-Fitr (24–26 May 2020), a comprehensive lockdown was implemented. This measure aimed to curtail the transmission of the virus, which was exacerbated by the customary practice of family gatherings during these occasions. Additionally, the lockdown was also enforced during the International Labor Day period, spanning from 1 May to 4 May 2020 (Government of Republic of Macedonia, 2020). The research conducted on personal protective equipment (PPE) among healthcare workers (HWs) revealed noteworthy findings. A significant proportion of participants, approximately 61.2%, reported the absence of isolation zones within their workplace. Additionally, a considerable number of HWs, around 33.4%, indicated that their workplace lacked a triage system for patients at the entrance. Furthermore, a substantial majority of HWs, approximately 72%, reported not having attended any training courses on the proper usage of PPE. This lack of training raises concerns about the potential for inadequate PPE utilization among healthcare workers. Moreover, a notable percentage of participants, approximately 25.7%, expressed uncertainty regarding the appropriate course of action following unwanted contact with blood or other secretions from a COVID-19 patient. This finding highlights the need for improved knowledge and guidance on post-exposure protocols among HWs. Overall, these findings shed light on several areas of concern regarding PPE practices among healthcare workers, including the absence of isolation zones, inadequate triage systems, insufficient training, and uncertainties surrounding post-exposure procedures. Addressing these issues is crucial to ensuring the safety and well-being of HWs in their efforts to combat COVID-19. It is worth mentioning that within the scope of this study, during the initial stages of the COVID-19 pandemic, a higher proportion of healthcare workers expressed dissatisfaction regarding the accessibility and adequacy of personal protective equipment (PPE) within their respective work environments (Mijakoski et al., 2020). The official List of Occupational Diseases (ODs) underwent a modification as of 07.05.2020, as documented in the Official Gazette, No 118/2020. The Ministry of Labor and Social Policy has recently made an important update to the List of occupational diseases. Specifically, they have included a new entry pertaining to infectious diseases caused by the coronavirus, specifically COVID-19. This inclusion applies to individuals who are engaged in various activities such as prevention, healthcare, home visits, or any other similar tasks that carry a proven risk of infection. To receive compensation for each of the occupational diseases (ODs) listed, the employee must possess the necessary “Expertise for the verification of occupational disease” issued by the Institute of Occupational Health of the Republic of North Macedonia. This document serves as a requirement for the verification process and subsequent compensation. Thus far, it has been observed that this optical device (OD) provides comprehensive coverage and compensation for all hardware components. The cases have the potential to be retrospectively confirmed as overdose incidents as the declaration of the COVID-19 pandemic by the World Health Organization (WHO) serves as a reference point (Rulebook on the List of Occupational Diseases, 2020). Compensation of health workers is as follows: The Institute of Occupational Health of R. North Macedonia verifies the occupational disease, the Commission for Work Ability Assessment (Medical Commission within the Pension Insurance Fund) confirms the verified occupational disease, and, finally, the Pension Insurance Fund compensates the affected health worker. In case of temporary work disability, the compensation is made by the Health Insurance Fund through their commission. Compensation mechanisms include treatment, rehabilitation, fully paid long-term sick leave, potential disability pension, and pension to surviving family members.

The results obtained for the Romanian sample of HWs (high participation rate, high degree of satisfaction with remuneration, and low levels of burnout dimensions) reflect the period of study realization (autumn of 2020) and the study subjects (high frequency of HWs with higher education and doctors), with a deep vocation allowing for the utilization of knowledge and experience accumulated in the profession, and their dedication, revealing the way of identification with the task of caring for patients with an agent etiologically less known than SARS-COV-2 was. The time chosen for the distribution of the questionnaire, in the autumn of 2020, was important because the study, as a method of development, behaved like a “mobilization campaign” of professionals in the health sector, counterbalancing the tendencies of “victimization” or direct “rewarding” through the financial compensatory mechanism. The approach offered by this study was supported, being perceived as a benefit by the HWs who were actively involved both in the care of COVID-19 patients (in COVID hospitals) and especially in the constant education of the population for protection, prevention, discipline, vaccination, and a healthy way of living and working. At the national policy level, the National Emergency Committee decided to maintain the disjunction between the management of the pandemic and the assessment of the specific working conditions in the health sector generated by that high level of uncertainty. At the beginning of 2020, through the establishment of the National Emergency Committee for the correct management of the pandemic, the medical body was used directly for the specific medical intervention for the patients but without holding the decision-making power at the macroeconomic level. This aspect had direct consequences on the professionals involved, especially as the visibility of the HWs to the public was increased by the media and the surveillance institutions. We note that at that moment, HWs were caught without protective equipment appropriate to the level of exposure risk (De Kock et al., 2021) and with a poor organization of the security and protection systems (OHS). The visibility of the health system professionals faced with the uncertainty of the pandemic phenomenon made them either the target of admiration “heroes” or the target of the violence, harassment, and aggression generated by the frustration due to the isolation and quarantine measures and the limitations to mobility or work. As a general conclusion, the results of the statistical processing of the data and information provided by the subjects involved (statistically significant for the predictors of burnout) mathematically support the strategic solutions for managing the pandemic, as described above. The behavior of the predictors of burnout in the “medium” or “low” level (without any “high” level) for the Romanian health sector supports the need to continue interventions to improve working conditions and promote “the health of the health workers,” as the 2021 conference and dissemination of the occupational health and safety guide issued by the WHO did (National Conference on Occupational Medicine, 2023).

HWs in Serbia, as an ambitious group, like challenges, and this had a significant impact on the Serbian results. It should be mentioned that most Serbian patients needed oxygen therapy and that Serbia does not have a central distribution of oxygen, so providing oxygen therapy was a physically demanding job (e.g., manual transport and distribution of oxygen cylinders). Therefore, this means that the most severe cases were not hospitalized at the Institute of Occupational Health (most respondents worked at the Institute) and that there were only a few fatal cases until the survey period. Since the respondents dealt mostly with patients who were successfully treated and released from the hospital in good condition, this also had an impact on the prevention of job burnout since invested efforts in the treatment of patients had a positive effect. Furthermore, it should be mentioned that HWs for the first time in the past 30–40 years were recognized as an important part of society and that there were several “positive” articles in the media during this period. For example, taxi drivers offered free rides to HWs. Most healthcare institutions responsible for the treatment of COVID-19 patients had 12 h shifts and work organized in a 12–24–12-48 regime. The other healthcare institutions reduced activity and from time to time sent their staff to COVID hospitals. A significant number of HWs were not fully engaged. HWs who were engaged in COVID institutions received around 30% increased salaries.

The pandemic demonstrated that the healthcare systems were not prepared to cope with the stress or unexpected situations. Therefore, it is essential to develop and adapt stress test models, similar to stress tests for banks. Stress tests for banks are a crucial supervision tool used to evaluate the resilience of the banking system to adverse, but plausible, future shocks. These tests can not only lead to regulatory changes but also influence the strategic decisions of the banks themselves. Stress tests have become a key part of banking supervision after the global financial crisis in 2008 (European Banking Authority, 2023; European Central Bank, 2023). This crisis exposed weaknesses in the abilities of banks to assess and manage risks, especially in stressful conditions, and our results showed that the pandemic imposed on the healthcare system even bigger crises, resulting in sometimes debilitating outcomes for the human capital in healthcare and, thus, in burnouts and psychiatric diseases. Similarly to way that the 2008 financial crisis resulted in the introduction of stress tests necessary for banks, we find that a modified stress test should be implemented in hospitals regarding future shocks that might include new pandemics, terrorism, catastrophes, or border conflicts.

The strength of this study is the large number of participating health workers. The participants are from 12 SEE countries. The survey was conducted in autumn 2020, after the first wave and during the second wave of the pandemic. This enabled us to receive data from an ongoing pandemic experience.

One of the limitations of the study is that it was impossible to assess the response rate. Since the questionnaire was sent via e-mail, links to the online questionnaires were available on the websites of the Medical and Nursing Chambers (for each of the participating countries) and through Microsoft Forms and LinkedIn, meaning it is impossible to assess how many health workers the invitation reached.

Another limitation is that the questionnaires used were self-administered questionnaires, which might have introduced recall bias. An unequal number of participants from each of the participating countries was another limitation. One more limitation is that the majority of the participants were female. This sex distribution was similar in the respective countries included in the survey. The age of the participants approximated the average age of the target population.

## 5. Conclusion

The current situation necessitates the prompt implementation of country-specific preventive measures aimed at mitigating burnout and enhancing work ability among healthcare workers (HWs) both during the ongoing pandemic and in the post-pandemic period. Preventive measures for psychosocial risks should be developed and applied, specifically for health workers. A modified stress test should be implemented in hospitals regarding future shocks that might include new pandemics, terrorism, catastrophes, or border conflicts.

## Data availability statement

The raw data supporting the conclusions of this article will be made available by the authors, without undue reservation.

## Ethics statement

The studies involving humans were approved by SEENWH and Ethical Boards of Institute of Occupational Health of RN Macedonia. The studies were conducted in accordance with the local legislation and institutional requirements. The participants provided their written informed consent to participate in this study.

## Author contributions

DM: Conceptualization, Formal analysis, Investigation, Methodology, Software, Supervision, Validation, Writing – original draft, Writing – review & editing. AA: Investigation, Validation, Writing – original draft, Writing – review & editing. DB: Investigation, Software, Validation, Writing – original draft, Writing – review & editing. HB: Formal analysis, Investigation, Validation, Writing – original draft, Writing – review & editing. OB: Formal analysis, Investigation, Validation, Writing – original draft, Writing – review & editing. LCK: Investigation, Validation, Writing – review & editing. MM: Formal analysis, Investigation, Validation, Writing – review & editing. JM: Data curation, Investigation, Validation, Writing – review & editing. BÖ: Investigation, Validation, Writing – review & editing. NP: Investigation, Validation, Writing – review & editing. LR: Investigation, Validation, Writing – review & editing. SS: Data curation, Investigation, Validation, Writing – review & editing. KV: Investigation, Validation, Writing – review & editing. RŽ: Investigation, Validation, Writing – review & editing. PB: Investigation, Validation, Writing – review & editing. AM: Investigation, Validation, Writing – review & editing. JK-B: Investigation, Validation, Writing – review & editing, Conceptualization, Methodology, Supervision.
